# Targeting NLRP3-Mediated Neuroinflammation in Alzheimer’s Disease Treatment

**DOI:** 10.3390/ijms23168979

**Published:** 2022-08-11

**Authors:** Julia Barczuk, Natalia Siwecka, Weronika Lusa, Wioletta Rozpędek-Kamińska, Ewa Kucharska, Ireneusz Majsterek

**Affiliations:** 1 Department of Clinical Chemistry and Biochemistry, Medical University of Lodz, 90-419 Lodz, Poland; 2 Department of Gerontology, Geriatrics and Social Work, Jesuit University Ignatianum, 31-501 Krakow, Poland

**Keywords:** Alzheimer’s disease, amyloid β, neurofibrillary tangles, NOD-like receptor pyrin domain-containing 3, NOD-like receptor pyrin domain-containing 3 inflammasome, NOD-like receptor pyrin domain-containing 3 inhibitors, Alzheimer’s disease treatment

## Abstract

Alzheimer’s disease (AD) is the most common cause of dementia in the general population and, to date, constitutes a major therapeutic challenge. In the pathogenesis of AD, aggregates of amyloid β (Aβ) and neurofibrillary tangles (NFTs) containing Tau-microtubule-associated protein (tau) are known to trigger a neuroinflammatory response with subsequent formation of an inflammasome. In particular, the NOD-like receptor pyrin domain-containing 3 (NLRP3) inflammasome is thought to play a crucial role in AD-related pathology. While the mechanisms for NLRP3 activation are not fully understood, it has been demonstrated that, after detection of protein aggregates, NLRP3 induces pro-inflammatory cytokines, such as interleukin 18 (IL-18) or interleukin 1β (IL-1β), that further potentiate AD progression. Specific inhibitors of NLRP3 that exhibit various mechanisms to attenuate the activity of NLRP3 have been tested in in vivo studies and have yielded promising results, as shown by the reduced level of tau and Aβ aggregates and diminished cognitive impairment. Herein, we would like to summarize the current state of knowledge on NLRP3 inflammasome priming, activation, and its actual role in AD pathogenesis, and to characterize the NLRP3 inhibitors that have been studied most and their impact on AD-related pathology.

## 1. Introduction

Alzheimer’s disease (AD) is the most common cause of dementia and is estimated to be at least the sixth leading killer in America [1]. In general, it is regarded as a complicated and multifaceted neurodegenerative disorder of the central nervous system (CNS). AD starts with a long preclinical or pre-symptomatic stage, which advances to mild cognitive impairment (MCI), and ultimately leads to dementia and incapacitating memory impairment. Besides the severity of AD, to date there is no cure for this disease and current treatment options remain only symptomatic [2,3].

Mechanistically, AD is characterized by an accumulation of amyloid plaques and neurofibrillary tangles (NFTs) that contribute to neuronal cell death and disease progression. The amyloid plaques are fibrous aggregates composed of Aβ. The abnormally folded Aβ is a by-product of the Amyloid Precursor Protein (APP) metabolism of several sequential proteases: β-secretase and the intramembranous γ-secretase complex [4,5,6]. Physiologically, the APP is processed by the α-secretase and γ-secretase, which does not produce Aβ. There are several types of soluble and insoluble non-plaque Aβ aggregates. Protofibrils and oligomers are the intermediate species that may form amyloid fibrils. There are also different variants of Aβ peptides that result from alternative processing, which makes them prone to exhibiting specific aggregation properties [7]. The Aβ(_1–40_) peptide is the most abundant isoform, whereas the Aβ(_1–42_) peptide is present in certain forms of AD [8,9]. On the other hand, the NFTs are composed of hyperphosphorylated Tau-microtubule-associated protein that accumulates within the dystrophic neurites. Interestingly, synapse loss, as well as the clinical features and the progression of AD, are better associated with NFT pathology than with β-amyloid pathology [4]. The cortical density of NFTs correlates better with cognitive decline than senile plaques [10]. Furthermore, the two antibodies targeting Aβ in a clinical trial did not manage to improve cognitive function despite decreasing Aβ burden, as revealed by PET imaging [11].

A crucial aspect of inflammatory responses is the formation of the inflammasome—a multiprotein complex that mediates inflammation. Amongst others, the NOD-like receptor pyrin domain-containing 3 (NLRP3) inflammasome is thought to play the greatest role in neurodegeneration, particularly in AD-associated pathology [12,13]. Although the mechanisms of its activation are not fully understood [14], many studies have demonstrated that the microglial NLRP3 inflammasome, due to its ability to sense aggregated proteins such as Aβ, alters the generation of interleukin 18 and interleukin 1β (IL-18 and IL-1β, respectively). These inflammatory mediators are closely associated with the spread of Aβ within and between the cerebral areas specific to AD pathogenesis, and thus contribute to disease progression [15,16,17]. Moreover, several studies have proven that inhibition of the NLRP3 inflammasome reduced tau aggregation and decreased the amyloid burden, which in turn diminished cognitive impairment in AD mice [18,19].

Due to the failure of many clinical trials investigating potential therapies targeting Aβ, it has been hypothesized that AD progression is due to a more global neuronal dysfunction [20]. Currently, it is believed that neuroinflammation has a huge impact on the development and progression of AD, opening up the opportunities for research into new possible drugs [21]. Considering the importance of neuroinflammation over the course of AD progression, this review summarizes current knowledge regarding NLRP3 inflammasome inhibition and strategies of its modulation that may contribute to the amelioration of this disease.

## 2. NLRP3 Inflammasome Activation and Stimulation

The NLRP3 inflammasome, first described in 2002 [22,23], is involved in the activation of caspase-1, leading to the release of IL-18 and IL-1β and resulting in a strong inflammatory response that has been observed in multiple diseases, including AD [24].

NLRP3 is one of the inflammasomes that has been studied most, and its molecular structure has already been determined. It contains a cytosolic sensor molecule NLRP3, ASC (the adaptor molecule apoptosis-associated speck-like protein containing a caspase recruitment domain [CARD]), and protease pro-caspase-1, which is the effector molecule [23]. The NLRP3 protein consists of a conserved central nucleotide binding and oligomerization domain (NOD or NACHT), C-terminal leucine-rich repeat (LRR) domain, and an N-terminal pyrin domain (PYD) [25]. NOD exhibits ATPase activity and is necessary for the self-oligomerization of the molecule at the beginning of the inflammasome’s assembly [26]. The role of the LRR domain is to recognize pathogen-associated molecular patterns (PAMPs), damage-associated molecular patterns (DAMPs), and other ligands, and to maintain the NLRP inactive state [27,28,29], whereas PYD allows communication with ASC through PYD–PYD domain interaction [30]. ASC recruits pro-caspase-1 through CARD–CARD interaction, which enables the release of inflammatory cytokines, such as IL-18 and IL-β [31]. Upon activation, the NLRP3 also executes a form of cell death (pyroptosis), which occurs via the induction of gasdemin D (GSDMD) secretion. It has been shown that members of the gasdemin family can bind to membrane lipids and induce membrane-disrupting cytotoxicity through the formation of pores in the cell membrane, which facilitates the secretion of inflammatory cytokines [32,33,34].

It is assumed that two steps are necessary for the activation of the NLRP3 inflammasome. The first step involves a primer signal that is associated with the nuclear factor kappa B (NF-κB)-dependent transcription of NLRP3 and pro-IL-1β [35]. A large number of receptors may be responsible for triggering this process, including anaphylatoxin, cytokine receptors, and pattern recognition receptors (PRRs) such as Toll-like receptors (TLRs) [36]. After binding, the specific receptor triggers the transcription of NLRP3 by activating NF-κB through different pathways that may be associated with myeloid differentiation primary response 88 (MyD88), TIR-domain-containing adapter-inducing interferon-β (TRIF), Fas-associated protein with death domain (FADD), or caspase-8, as well as with reactive oxygen species (ROS) [35,36,37].

**Figure 1:**
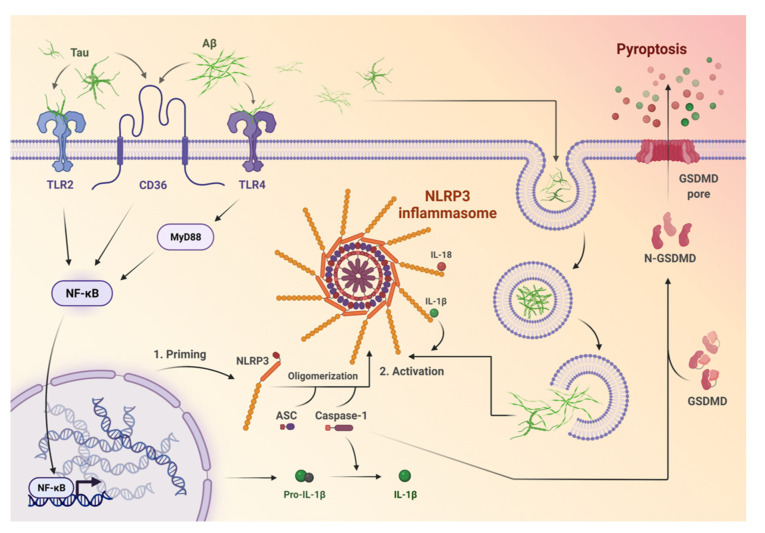
The mechanisms of priming and activation of the NOD-like receptor pyrin domain-containing 3 (NLRP3) inflammasome. Firstly, protein aggregates (Aβ and tau) are recognized by microglial pattern recognition receptors, such as Toll-like receptors (e.g., TLR2, TLR4), or cluster of differentiation 36 (CD36). Afterwards, they induce activation of specific pathways like MYD88/NF-κB, which leads to transcription of pro-IL-β and NLRP3. One of the NLRP3 domains—the adaptor molecule apoptosis-associated speck-like protein containing a caspase recruitment domain (ASC)—recruits pro-caspase-1, which enables the release of inflammatory cytokines, namely IL-18 and IL-β. Further, cell death in the form of pyroptosis occurs after the activation of NLRP3 via the induction of gasdemin D (GSDMD). GSDMD can induce membrane-disrupting cytotoxicity and facilitates the secretion of inflammatory cytokines via the creation of pores in the cell membrane.

The second stage involves the activation and assembly of the NLRP3 inflammasome, which can be induced by many factors that are related to the disturbance of cell homeostasis [38]. Although the exact mechanism by which NLRP3 activation occurs is still unknown, there are several activation signals that are suggested to promote the second step of NLRP3 formation. One of these activation signals involves molecules such as silica, Aβ, and crystalline substances that can be phagocytized by cells such as microglia but are resistant to degradation [39,40,41,42,43,44,45]. This resistance later causes the destabilization of lysosomes, which leads to the release of various enzymes, such as cathepsins, into the cytosol [39,46,47]. This process drives the rise of cytosolic Ca^2+^ levels, which is involved in the activation of NLRP3. Moreover, it has been shown that a high extracellular K^+^ concentration decreases the rise in intracellular Ca^2+^ levels. Due to this fact, K^+^ efflux could promote Ca^2+^ influx and increase cytosolic Ca^2+^ concentrations, leading to NLRP3 activation [36,48,49] (Figure 1).

Other circumstances that promote NLRP3 activation are ultraviolet radiation, the release of ATP by necrotic cells located nearby, or certain events that are related to mitochondrial damage, such as the loss of membrane potential, ROS production, the release of mitochondrial DNA (mtDNA), and the exposure of cardiolipin at the mitochondrial outer membrane. The latter is particularly and directly associated with NLRP3 activation [14,36,50,51,52]. The variety of mechanisms inducing NLRP3 illustrates that inflammasome activation is generally driven by the detection of homeostatic imbalance, not by a single pathway. 

Recently, it has been shown that human monocytes can form the NLRP3 inflammasome, bypassing the priming step that has long been viewed as essential. Numerous studies have also emphasized the role of transcription-independent priming in relation to post-translational modifications (PTMs) of NLRP3, such as ubiquitination and phosphorylation [53,54]. These changes are known to exert a significant impact on inflammasome functionality [55,56,57]. The role of other PTMs, such as methylation or acetylation, has not yet been thoroughly investigated, however the possibility of their involvement in NLRP3 activation cannot be excluded. It has also been suggested that, although priming may not always be obligatory for assembling a functional NLRP3 inflammasome, it may be required to boost inflammasome activation and the overall inflammation process [58].

## 3. NLRP3 Inflammasome in AD

NLRP3 inflammasome activation has been identified in AD patients by increased IL-1β and active caspase-1 production [19,59]. Transgenic animal models of AD, such as APP/PS1 mice [19] or Tg2576 mice [60], have also demonstrated NLRP3 activation through the presence of increased IL-1β and caspase-1 levels. Furthermore, the monocytes of patients with AD that were LPS-primed and Aβ_42_-stimulated have shown an increased expression of NLRP3 inflammasome components such as NLRP3, PYCARD, caspase-1, and its effectors: IL-18 and IL-1β [61]. Heneka et al. have found that AD mice with a double knockout of Nlrp3 or Casp1 (APP/PS1/NLRP3^−/−^ and APP/PS1/Casp-1^−/−^) demonstrate improved memory function, as well as decreased levels of caspase-1, Il-1β, and Aβ deposits compared to APP/PS1 mice. However, contrary to the previous reports, a new study by Tang et al. that investigated specific NLRP3 inflammasome markers in post-mortem brain tissues indicated that NLRP3 activation may in fact not take place in AD patients’ brains [62]. Thus, the actual effect of NLRP3 activation on the AD course is yet to be determined.

Of note, it has been shown that the AD-specific aggregates, Aβ and NFTs, are able to induce NLRP3 inflammasome priming through a number of mechanisms [63,64,65,66]. Aβ and tau are mostly recognized by microglial pattern recognition receptors, such as Toll-like receptors (e.g., TL2, TLR4), cluster of differentiation 14 (CD14), cluster of differentiation 47 (CD47), cluster of differentiation 36 (CD36), and α6β1 integrin. Then, they induce inflammation through specific pathways such as MYD88 or NF-κB [67,68], leading to the transcription of pro-IL-β and NLRP3 [38,69].

**Figure 2:**
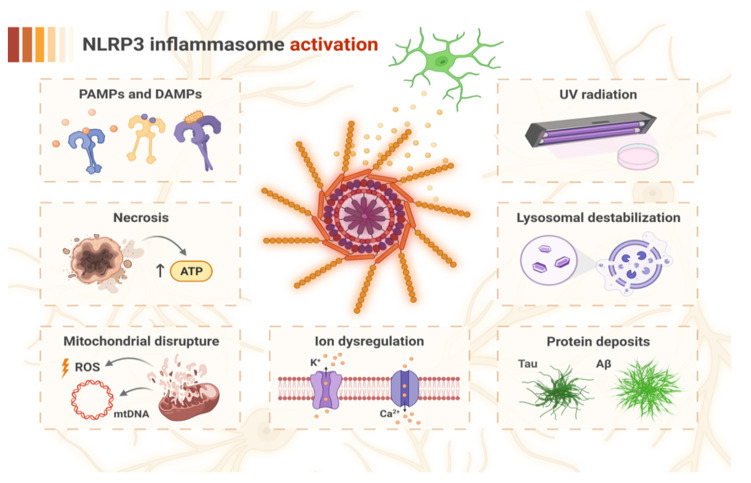
The factors inducing NOD-like receptor pyrin domain-containing 3 (NLRP3) inflammasome activation in the pathogenesis of AD. The recognition of pathogen-associated molecular patterns (PAMPs) and damage-associated molecular patterns (DAMPs) by specific cellular receptors may bring NLRP3 into the active state. Activation may also be triggered by factors related to cell damage, which involve mitochondrial disruption with the overproduction of reactive oxygen species (ROS) and mitochondrial DNA (mtDNA) release, necrosis with an increased level of ATP released from damaged cells, ion dysregulation (K^+^ efflux and Ca^2+^ influx), and lysosomal destabilization resulting from impaired microglial phagocytosis. Furthermore, protein deposits of Tau and amyloid β (Aβ) may drive lysosomal dysfunction due to their resistance to degradation, and may also directly induce NLRP3 activation. Additionally, UV radiation constitutes another independent factor for activation of the NLRP3 inflammasome.

The second step of NLRP3 inflammasome activation in AD may occur through lysosomal damage, the release of enzymes, such as cathepsin B, and the overproduction of mitochondrial ROS processes described above [50,70]. These events lead to the production of IL-1β, caspase 1 activation, pyroptosis, and neuroinflammation. Increased IL-1β and caspase-1 levels have been observed in patients with early or mild stages of AD, which suggests that NLRP3 inflammasome activation may contribute to early pathogenic events that stimulate the progression of AD [19,67,71,72,73]. APP/PS1 mice that are deficient in caspase-1 or NLRP3 have been shown to exhibit reduced spatial memory loss, decreased impairment of hippocampal synaptic plasticity, and amelioration of other AD-related symptoms [21,40] (Figure 2).

## 4. Neuroinflammation in the Pathogenesis and Progression of AD

Numerous diseases linked to immune system dysfunction and the overactivation of NLRP3 have been recognized as risk factors for developing AD. These disorders include autoimmune rheumatic diseases [74], traumatic brain injury [75], type 2 diabetes mellitus [76], obesity [77], cerebrovascular diseases [78], dyslipidemia [79], and hypertension [80,81]. The latter is strongly related to atherosclerosis, which is both an inflammatory disease involving NLRP3 activation and a vascular disease that may promote AD development by chronic hypoperfusion and the stiffening of arterial walls in the brain [80]. Hypertension is associated with increased Aβ and NFT deposits, and it has been established that midlife stage 1 and stage 2 systolic hypertension increases the risk of developing AD by 18 and 25%, respectively [81]. Aging, as the greatest risk factor for developing AD, is responsible for more than 95% cases of late-onset Alzheimer’s disease (LOAD). Senescence is strongly linked to immune system dysfunction: it may promote autoimmunity or constitutive low-grade inflammation, which is characteristic for AD. It was also found that different cell types, including astrocytes and microglia, present the accumulation of senescence-associated proteins, such as p16, p53, and p21 in AD patients’ brains, which impairs their proper functioning [82,83].

The NLRP3 inflammasome is present in microglia and astrocytes in the CNS [84,85,86]. Several studies have indicated that it can also be expressed in neurons and oligodendrocytes [87,88].

Microglia, which are macrophages found in the CNS, are crucial in maintaining brain homeostasis throughout life. They are involved in the innate immune response as a first-line defense against invading pathogens. Not so long ago, microglia were shown to exhibit the capacity to develop innate immune memory (IIM), which has changed our perspective on inborn immunity. The IIM indicates that microglial activity can be molecularly reprogrammed, leading to the suppression (immune tolerance characteristic of the M2 microglial phenotype) or enhancement of the immune response (trained immunity concerning the M1 phenotype) [89,90,91]. Similarly to microglia, astrocytes can also be divided into two phenotypes: the harmful A1 cells and the protective A2 cells, which express neurotrophic factors such as brain derived neurotrophic factor (BDNF) [92]. This dichotomic classification, although simplified, is still useful for conveying the idea of the specific nature of glial cells, depending on the circumstances [69].

There is growing evidence to suggest that microglial cells, upon activation, may lose their ability to retransform to a naïve status. Additionally, they are thought to remain “post-activated”, which may lead to some neuropathological changes [93]. IIM may exert an impact on the progression of neurodegeneration through an enhanced immune response and the polarization of microglia to the more active M1 phenotype. Moreover, it was shown that polarization to the immune-suppressive M2 phenotype can reduce neuroinflammation and presumably ameliorate neurodegeneration [94,95,96,97].

Genome studies have allowed for the detection of more than 40 gene variants that are associated with the development of late-onset AD [98]. Among these, there are some genes that are related to the mediation of inflammation, particularly microglial immunoreceptors such as TREM2, PILRA, and CD33. TREM2 is responsible for the enhancement of microglial phagocytosis and the secretion of inflammatory cytokines in response to specific stimuli, involving NFTs and Aβ [99]. It is important to note that, during the primal stages of AD, a lack of TREM2 ameliorates amyloid pathology and synaptic deficit, whereas it has the opposite affect at later stages (a detrimental effect due to the absence of Aβ phagocytosis) [100,101]. This conclusion is consistent with the two-peak theory of microglial activation in the progression of AD. In this theory, the first peak involves the phagocytosis of Aβ by the protective M2 phenotype and the second peak is driven by NFTs that activate the pro-inflammatory M1 phenotype [102,103].

The dual nature of neuroinflammation in AD suggests that an approach based simply on suppressing glial function may in fact be daunting. A more rational treatment option should probably focus on analyzing whether the regions of a patient’s brain are in the early or late stages of AD, which could help to distinguish between the neurotoxic or neuroprotective phenotypes of microglia [102]. In this case, selective inhibition of the more reactive and neurotoxic microglial phenotype should also be considered.

## 5. NLRP3 Inhibitors

Many preclinical models imply that small-molecule NLRP3 inhibitors may be practically useful against numerous diseases that involve neuroinflammation. In contrast to antibodies or vaccines, they possess the unquestionable advantage of being able to cross the brain blood barrier (BBB), which has been exploited in a number of studies of neurodegenerative disorders and other pathologies related to the CNS [104,105,106] (Table 1).

### 5.1. MCC950

MCC950 (1-(1,2,3,5,6,7-Hexahydro-s-indacen-4-yl)-3-[4-(1-hydroxy-1-methyl-ethyl)-furan-2-sulfonyl]urea, also known as CRID3 and CP-456773) is one of the small-molecule inhibitors of the NLRP3 inflammasome. MCC950 interacts with the Walker B motif of the NLRP3 NACHT domain, that is, the Mg^2+^-binding ATPase specific P-loop. This interaction results in the blocking of ATP hydrolysis, the inhibition of NLRP3 activation, and oligomerization [104,107,108,109]. Studies on APP/PS1 mouse models of AD have shown that MCC950 is able to ameliorate cognitive function, presumably due to the drug’s ability reduce Aβ accumulation by stimulating its phagocytosis in vitro [110].

MCC950 has also been shown to inhibit microglial training induced by NLRP3 stimulation in sporadic AD mouse models. Therefore, it protects against pathologies such as Aβ accumulation or neuronal loss [111]. Another study demonstrated that MCC950 inhibits neuronal pyroptosis and significantly reduces Aβ neurotoxicity in human primary neurons in vitro. The treatment of AD mice with MCC950 positively impacted the histological morphology of senescence-accelerated mouse prone 8 (SAMP8) mice brains and improved the spatial memory of treated animals. Additionally, MCC950 reduced the overexpression of GSDMD, caspase-1, and NLRP3-response factors, which are strongly involved in neuronal pyroptosis in SAMP8 mice [112]. Although MCC950 exhibits high target selectivity, its therapeutic development is limited by its toxicokinetic properties [82]. It has been suggested that MCC950 could have unwanted effects by inhibiting carbonic anhydrase 2 and other carbonic anhydrases, which are widely expressed in human cells and involved in maintaining fluid balance and pH levels. For that reason, the focus has been on the development of less toxic MCC950 analogues, such as OLT1177, which will be described later in this review [113]. Despite this, MCC950 and Inzomelid (a related NLRP3 inhibitor), have now passed through phase 1b clinical trials and will soon move to phase 2 for a range of CNS degenerative disorders, such as AD or PD [114].

### 5.2. JC124

JC124 (5-chloro-2-methoxy-N-(4-(N-methylsulfamoyl)phenethyl)benzamide) is another small-molecule inhibitor of the NLRP3 inflammasome, which functions by blocking caspase-1 activation, IL-1β secretion, and ASC aggregation [115]. JC124 has been shown to protect mice brains from excessive inflammation after a traumatic brain injury (TBI). Post-injury treatment with the drug leads to reduced neuronal degeneration and inflammatory cell responses, as well as a decreased expression of molecules such as NLRP3, ASC, caspase-1, and IL-1β [116]. Moreover, JC124 has been shown to reduce Aβ load and neuroinflammation in APP/PS1 mice, leading to ameliorated synaptic plasticity and an improvement in cognitive function [117]. Another study has examined the effects of JC124 on AD-related pathologies in CRND8 APP transgenic mice (TgCRND8). JC124 treatment reduced the levels of both soluble and insoluble Aβ, decreased β-cleavage of APP, and increased astrocytosis, all whilst decreasing microglial activity at the same time. JC124 also reduces oxidative stress, indicating its neuroprotective effects. Moreover, the same study showed that even at a later point of amyloidosis progression, inhibition of the NLRP3 inflammasome can efficiently reduce the effects of amyloid accumulation and oxidative stress [118]. The above-mentioned findings make JC124 and its analogues promising drug candidates for the potential treatment of AD.

### 5.3. VX-765

VX-765 (N-(4-Amino-3-chlorobenzoyl)-3-methyl-L-valyl-N-[(2R,3S)-2-ethoxytetrahydro-5-oxo-3-furanyl]-L-prolinamide, also known as Belnacasan) is a small caspase-1 and caspase-4 inhibitor. Caspase-1-mediated inflammation is strongly involved in the activation and functioning of the NLRP3 inflammasome and is believed to play a crucial role in the onset of AD. VX-765 is BBB permeable, nontoxic, and has already been approved by the Food and Drug Administration (FDA) for clinical trials involving humans. This molecule has displayed the ability to reverse episodic and spatial memory impairment in a J20 mouse model by halting the progression of Aβ deposition. It also managed to reverse neuroinflammation and stabilize synaptophysin protein levels in the mouse hippocampus [119]. Another study has demonstrated that, in Swedish/Indiana mutant amyloid precursor protein (APPSw/Ind) J20 and wild-type mice, VX-765 postponed either AD- or age-related spatial memory deficits after pre-symptomatic treatment [120]. VX-765 was also studied in the context of neuroinflammation occurring after spinal cord injury (SCI) in mice, where it was shown to inhibit the secretion of caspase-1, IL-1β, and IL-18. Moreover, VX-765 decreased total macrophage infiltration, Th1 and Th17 differentiation, and M1 microglia activation, while promoting the differentiation of type 2 helper T cells (Th2) and regulatory T cells (Treg) and the activation of M2 microglia. Further, administration of the drug led to the alleviation of neural injury and white matter demyelination, thereby improving functional recovery from the trauma [121]. In rat models of temporal lobe epilepsy or stroke, VX-765 was also shown to decrease pyroptosis in cerebral ischemia and was shown to have an overall neuroprotective effect [122,123,124]. 

### 5.4. OLT1177

OLT1177 (3-(Methane sulfonyl)propanenitrile, also known as Dapansutrile) is an orally active β-sulfonyl nitrile molecule and a specific inhibitor of the NLRP3 inflammasome that has been proven to be safe for humans. Mechanistically, it inhibits the properties of NLRP3 that are associated with ATPase activity that is vital for ASC recruitment, thus preventing NLRP3-ASC formation and inflammasome activation. Besides inhibiting NLRPR3-ASC, OLT1177 also affects NLRP3-caspase-1 interaction. In vitro, OLT117 reduced the release of IL-1β and IL-18 resulting from NLRP3 inflammasome activation. In vitro, the inhibitor reduced IL-1β levels at concentrations more than 100-fold lower than the effective plasma concentrations of IL-1β that have been safely reached in humans. Moreover, it was shown that OLT1177 inhibits the accumulation of succinate in muscles, the level of which is correlated with oxidative stress driving mitochondrial uncoupling [125]. OLT117 was also studied in mouse models of multiple sclerosis (MS), wherein it improved the disease symptoms by reducing the infiltration of macrophages and CD4 T cells in the spinal cord, as well as decreasing levels of IL-1β and IL-18 [126]. In APP/PS1 mice, OLT1177 ameliorated synaptic plasticity, inhibited the activation of microglia, and decreased the number of Aβ plaques in the cortex. Furthermore, the study has shown that the levels of metabolic markers of AD (e.g., carboxylic acids, 5-oxoproline, kynurenine deaminated purines, glutaminolysis) in plasma were restored, depending on the administered dose of OLT117 [127].

### 5.5. NSAIDs

Recently, it has been suggested that nonsteroidal anti-inflammatory drugs (NSAIDs), due to their anti-inflammatory characteristics, may also inhibit neuroinflammation occurring at the onset of AD, and hence they may play a role in decreasing the risk of developing AD [128,129,130,131]. NSAIDs are one of the most commonly used medications and they appear on the World Health Organization (WHO) Model List of Essential Medicines. NSAIDs inhibit the cyclooxygenase-1/2 (COX-1 and COX-2) enzymes and are also able to selectively inhibit the NLRP3 inflammasome by interacting with volume-regulated anion channels independently of COX enzymes [132,133,134,135]. However, placebo-controlled clinical trials with NSAIDs in AD patients have shown negative outcomes. Neither rofecoxib in patients with MCI, nor naproxen and celecoxib administered to elderly patients have exhibited positive results [136]. Even though some studies have demonstrated a reduced risk of AD mortality with aspirin or other NSAIDs [137], which supports the hypothesis of the neuroprotective role of NSAIDs in AD development, thorough studies and meta-analyses indicate that there is no evidence to encourage the use of NSAIDs of any class for the prevention of dementia [136,138,139]. Furthermore, studies also highlight the adverse health effects that may result from sustained treatment with NSAIDs [140], for example, gastric irritation or the higher risk of ulcer formation [141], that are triggered by selective COX-1 inhibition.

### 5.6. Colchicine

Interestingly, colchicine, an alkaloid derived from autumn crocus (*Colchicum autumnale*), has already been shown to inhibit NLRP3 inflammasome oligomerization in patients with atherosclerosis [142,143]. However, the drug was able to induce AD-like symptoms, such as dementia or cognitive impairment, in rodents by single intracerebroventricular injection, as evaluated through the use of Morris water maze and elevated plus-maze tests [142,144,145]. This could be explained by colchicine’s ability to promote neuroinflammation via the activation of a number of other pathways and effectors, such as COX-2 [146,147], TNF-α, nitrite, or ROS in the hippocampus [148]. Moreover, it was found that colchicine directly destroys hippocampal granule cells and mossy fibers via the blockage of axoplasmic transport, which eventually leads to cholinergic neurodegeneration and AD symptoms [149].

## 6. Conclusions

It is clear that the NLRP3/caspase-1/IL-1β axis is involved in crucial pathological events that are associated with AD. According to the latest research, it is the neuroinflammatory response to amyloid plaques that triggers the development and progression of AD, not the presence of the aggregates themselves. This review summarizes our current understanding of the NLRP3 inflammasome, the processes of its activation and stimulation, and its involvement in AD pathogenesis and neuroinflammation. We also reviewed the most popular NLRP3 inhibitors, which exhibit different mechanisms of attenuating NLRP3 activity and decreasing inflammation. MCC950 and OLT1177 block the ATPase activity that is essential for NLRP3 activation, and JC124 inhibits caspase-1 activation, IL-1β secretion, and ASC aggregation, whereas VX-765 prevents the activation of caspases 1 and 4. However, many important questions prevail, for example, which disease stage inhibitors of NLRP3 may be useful, or what mechanism of their action is most beneficial for ameliorating the progression of AD? AD is a complex disorder and so much remains to be explored in order to deepen our understanding of the complexity of neuroinflammation in CNS pathologies. The phenotypic changes taking place in the glial cells during the pathogenesis of these diseases remain particularly intriguing, as well as the possibility to target the innate immune stem cells and the IIM. Considering the positive findings regarding the amelioration of cognitive function in animal models that were treated with NLRP3 inflammasome inhibitors, as well as the first attempts to undertake clinical trials with the afore-mentioned drugs, this area of research sheds a light on the development of new potential approaches to AD treatment. However, there is still much to be investigated before efficient drugs that inhibit NLRP3 are officially approved for the treatment of AD. Nevertheless, the modulation of neuroinflammation remains a promising target of potential molecular therapy for this incurable disease.

## Figures and Tables

**Table 1:** Characterization of the most common NLRP3 inhibitors used in AD research.

Compound Code of Inhibitor	Chemical Name ofInhibitor	Chemical Structure of Inhibitor	Mechanism of Action	Research Models
**MCC950** (also known as CRID-3 or CP-456773)	1-(1,2,3,5,6,7-Hexahydro-*s*-indacen-4-yl)-3-[4-(1-hydroxy-1-methyl-ethyl)-furan-2-sulfonyl]urea	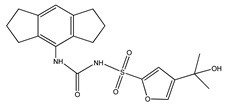	inhibition of NACHT domain of NLRP3	- APP/PS1 AD mouse model [110] - sporadic AD mouse model [111] - SAMP8 mouse model [112]
**JC124**	(5-Chloro-2-methoxy-N-(4-(N-methylsulfamoyl) phenethyl)benzamide	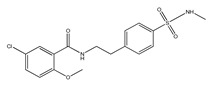	inhibition of caspase-1 activation, IL-1β secretion, and ASC aggregation	- APP/PS1 AD mouse model [117] - TgCRND8 AD mouse model [118]
**VX-765** (also known as Belnacasan)	*N*-(4-Amino-3-chlorobenzoyl)-3-methyl-L-valyl-*N*-[(2*R*,3*S*)-2-ethoxytetrahydro-5-oxo-3-furanyl]-L-prolinamide	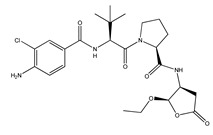	inhibition of caspase-1 and caspase-4	- J20 AD mouse model [119] - APP^Sw/Ind^ J20 and wild-type AD mouse model [120]
**OLT1177** (also known as Dapansutrile)	3-(Methane sulfonyl)propanenitrile	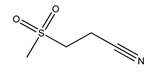	inhibition of NLRP-ASC and NLRP3-caspase-1 interaction	- APP/PS1 AD mouse model [127]

## Data Availability

Not applicable.
